# Countermovement Jump Inter-Limb Asymmetries in Collegiate Basketball Players

**DOI:** 10.3390/sports7050103

**Published:** 2019-04-30

**Authors:** Aaron Heishman, Bryce Daub, Ryan Miller, Brady Brown, Eduardo Freitas, Michael Bemben

**Affiliations:** 1 Department of Health and Exercise Science, University of Oklahoma, Norman, OK 73019, USA; ryanmiller1@ou.edu (R.M.); brownbrady3@ou.edu (B.B.); eduardofreitas@ou.edu (E.F.); mgbemben@ou.edu (M.B.); 2 Department of Athletics, Basketball Strength and Performance, University of Oklahoma, Norman, OK 73019 USA; bdaub@ou.edu

**Keywords:** athlete monitoring, athlete performance, reliability, fatigue monitoring, bilateral countermovement jump, CMJ arm swing, CMJ without arm swing

## Abstract

The purpose of the present study was to establish the intrasession and intersession reliability of variables obtained from a force plate that was used to quantitate lower extremity inter-limb asymmetry during the bilateral countermovement jump (CMJ). Secondarily, a comparison was performed to determine the influence of the jump protocol CMJ with or without an arm swing (CMJ AS and CMJ NAS, respectively) on inter-limb asymmetries. Twenty-two collegiate basketball players performed three CMJ AS and three CMJ NAS on dual force platforms during two separate testing sessions. A majority of variables met the acceptable criterion of intersession and intrasession relative reliability (ICC > 0.700), while fewer than half met standards established for absolute reliability (CV < 10%). CMJ protocol appeared to influence asymmetries; Concentric Impulse-100 ms, Eccentric Braking Rate of Force Development, Eccentric Deceleration, and Force at Zero velocity were significantly different between jumping conditions (CMJAS versus CMJ NAS; *p* < 0.05). The present data establish the reliability and smallest worthwhile change of inter-limb asymmetries during the CMJ, while also identifying the influence of CMJ protocol on inter-limb asymmetries, which can be useful to practitioners and clinicians in order to effectively monitor changes associated with performance, injury risk, and return-to-play strategies.

## 1. Introduction

Assessing and monitoring mechanical inter-limb differences, or asymmetries, has become an increasingly common practice among researchers, coaches, and clinicians in an effort to provide insight into athletic performance, injury prevention, and rehabilitation. More specifically, increases in lower-limb power asymmetries have been associated with decreases in jump height and sprint speed, as well as reductions in change-of-direction speeds [1,2,3]. Similarly, asymmetries in movement patterns have been proposed as a factor increasing injury risk [4,5,6]. Inter-limb asymmetry measurements have also been found to be useful in quantitating functional deficits following an injury, and when guiding an athlete’s readiness to return-to-play and later return-to-performance as part of the rehabilitation process [7,8,9]. The continued advancement of viable field tests that are non-invasive and time-efficient is essential in order for practitioners and clinicians to frequently monitor changes in inter-limb asymmetries that may be detrimental to performance or place athletes at an elevated injury risk. 

A variety of methods have been used to assess lower-limb force and strength asymmetries [10,11]. The isokinetic force test is the most common means of testing asymmetries; however, the arthrokinematics and contraction speed of isokinetic testing vastly differ from those encountered during sport [12]. Previous literature noted the inability of isokinetic assessments to identify bilateral symmetries alone, with more sport-specific assessments required to reveal asymmetries [13]. Moreover, isokinetic testing requires expensive equipment and lacks the time efficiency required for frequent assessments of numerous athletes in the team-sport setting. Limitations associated with isokinetic testing have steered the development of more functional assessments to measure lower extremity asymmetries, including the single-leg countermovement jump (SLCMJ), as well as the bilateral countermovement jump (CMJ) [10,12,14]. 

In contrast to isokinetic testing, the SLCMJ and CMJ offers a functional assessment paralleling the dynamic, closed-chain movement experienced in sport, while also incorporating the stretch-shortening cycle (SSC) [12,15]. Although the SLCMJ does offer an advantage over isokinetic testing as a dynamic functional assessment, it is not without limitations, as performance can be influenced by additional factors, such as movement coordination and balance [12,16]. These factors have led to a closer examination of the utility of the CMJ. The CMJ is commonly used in the applied performance setting to monitor acute changes in neuromuscular readiness and fatigue, while also used to assess long-term changes in performance qualities, such as adaptations to a training program [17,18,19,20]. Previously, Impellizzeri et al. [12] developed an assessment to quantify asymmetries in peak vertical ground reaction forces during the CMJ, where athletes jump with one foot placed on a force plate and the other on a wooden platform to isolate the individual limb contributions during the jump. However, the CMJ is increasingly being performed on dual force plates, with each lower limb positioned on an individual force platform. The dual force platforms can be synchronized to provide the conventional analysis of bilateral actions during the CMJ [21], while also simultaneously monitoring the force-time signature of each individual limb, allowing the delineation of lower-limb force contributions and asymmetries. 

The CMJ involves the dynamic muscle function known as the stretch-shortening cycle (SSC) [15]. Although the SSC requires both concentric and eccentric muscle actions, previous research investigating lower-limb asymmetries has only focused on variables revolving around the concentric phase (i.e., force, power, etc.), neglecting the performance of the eccentric portion [19,21]. However, examining the characteristics of the eccentric phase may offer novel insights associated with changes in neuromuscular function and movement strategies during the CMJ [22]. Therefore, establishing the reliability of lower-limb asymmetry throughout the entire CMJ force-time signature may be critical for performance practitioners and clinicians to effectively monitor changes associated with performance, injury risk, and return-to-play strategies. 

Two methods are commonly employed when performing the CMJ. The first approach incorporates the use of the arm swing (CMJ AS), and has been suggested to facilitate a higher level of sport-specificity, as well as enhance performance during the CMJ [21,23,24]. However, it is speculated that the arm swing may counteract lower extremity actions and mask lower limb force asymmetries. In contrast, the second approach eliminates the influence of the arm swing by requiring the athletes to maintain hand placement on the hips or fixed to a virtually weightless implement (e.g., a polyvinyl chloride pipe or wooden dowel) positioned on their shoulders throughout the duration of the CMJ (CMJ NAS) [12,13,18,21]. Proponents of this protocol suggest that the elimination of the arm swing isolates lower extremity function and reduces the potential variability introduced by performing the arm swing movement. However, the unfamiliar nature of eliminating the arm swing during the CMJ has been speculated to modify movement strategies, especially in athletes participating in sports that involve substantial amounts of jumping. In addition, it is speculated the arm swing may counteract lower extremity action and mask lower limb force asymmetries. While the reliability of the CMJ has been established when lower-extremity outputs are assessed in conjunction [18,19,21], to our knowledge, no evidence exists that specifically identifies the influence of the CMJ AS and CMJ NAS on the reliability of inter-limb asymmetries during the bilateral CMJ. 

Previous literature has consistently considered an asymmetry greater than 10% between limbs as clinically relevant, and this threshold has been used as a criterion to guide return-to-play following injury [7,8,11]. However, the lack of data establishing the reliability and smallest worthwhile change in inter-limb asymmetry may make the traditional 10% rule unqualified, as it would not allow the separation of the “signal” from the “noise” [25]. While research has established the reliability of the CMJ [18,21], the reliability and the smallest worthwhile change of lower extremity inter-limb asymmetries during the CMJ remains to be explored. Further, although both CMJ protocols (CMJ AS and CMJ NAS) have been used to evaluate lower extremity inter-limb asymmetries [12,13,16,24], to our knowledge, previous research has yet to identify the influence of the CMJ protocol, with or without the arm swing, on the reliability of lower extremity inter-limb asymmetries. Therefore, the primary purpose of the present investigation was to establish the intersession and intrasession relative and absolute reliability of variables obtained from a force plate used to quantitate lower extremity inter-limb asymmetry during both the CMJ AS and CMJ NAS in a cohort of healthy collegiate basketball players. A secondary purpose was to examine inter-limb symmetry between the CMJ AS compared to the CMJ NAS. It was hypothesized that both CMJ protocols would meet acceptable standards of relative and absolute reliability. Additionally, it was hypothesized that there would be differences in asymmetries between jump protocols, with CMJ AS demonstrating a reduction in between limb asymmetries. 

## 2. Materials and Methods

### 2.1. Subjects

A convenience sample of 22 (Men: n = 14, age = 19.7 ± 1.0 years, height = 1.98 ± 0.71 m, body mass = 94.7 ± 6.2 kg; Women: n = 8, age = 20 ± 1.6 years, height = 1.80 ± 0.65 m, body mass = 78.2 ± 8.3 kg) National Collegiate Athletic Association (NCAA) Division 1 collegiate basketball players were included in this study. All the subjects were active squad members of the University of Oklahoma’s Men’s and Women’s Basketball teams and were free of any acute musculoskeletal injuries at the time of testing. This research was approved by the Institutional Review Board of the University of Oklahoma, and all the subjects provided written, informed consent before participating in the study. In addition, this study conforms to the standards set by the Declaration of Helsinki.

### 2.2. Design

**Figure 1:**
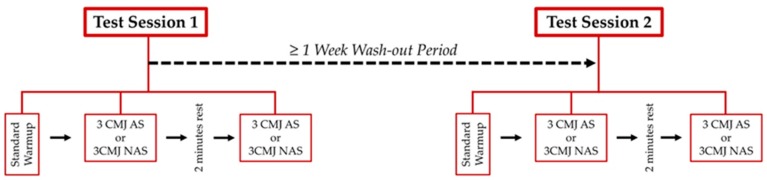
The study design and flow of testing. During Test Session 1, subjects performed either three CMJ AS or three CMJ NAS in a randomized order, followed by three jumps of the other protocol. During Test Session 2, subjects performed the same number of jumps in the reciprocal order. CMJ represents countermovement jump; AS represents arm swing; NAS represents no arm swing.

A randomized cross-over within-subject study design was utilized to examine the reliability of the side-to-side limb symmetry of CMJ variables during both the CMJ AS and the CMJ NAS. As illustrated in Figure 1, during two different testing sessions, which were separated by at least one week, subjects performed three CMJs with an arm swing and three CMJs with no arm swing, in a randomized order. 

### 2.3. Procedures

All the testing took place within a two-week time frame during the off-season training period. Subjects performed two testing sessions, with both sessions including three CMJ AS and three CMJ NAS. The order of jump type was randomly assigned during Test Session 1, and subjects performed the jump type in the reciprocal order during Test Session 2. A minimum of two minutes of rest was allotted between jump trials. In addition, each subject performed both testing sessions within the same hour, and all the subjects performed their testing in the afternoon between 13:00 and 14:00, as previous literature has identified the influence of time of day on jump performance [26]. In accordance with prior literature [18,21] and in an attempt to control the impact of training loads on testing outcomes, CMJ testing was performed within the same time frame of the training week, and training loads were strictly matched 72 h prior to both testing sessions, with sport-specific practice duration matched in the days prior to both testing sessions. Further, subjects were instructed to have no physical exertion or exercise prior to arriving on days of testing. In an effort to maintain ecological validity, subjects wore their standard practice gear, including shoes of their choosing, but each subject was required to wear the same pair of shoes during both testing sessions. While no dietary restrictions were implemented, athletes were instructed to maintain normal dietary intake, as outlined by the team’s sports nutritionist. To limit the impact of instructions on the CMJ performance characteristics, consistent instructions were provided to all the subjects during each CMJ trial. In addition, verbal encouragement was provided to encourage maximal effort during each jump attempt. 

All the testing was conducted at the basketball training facility prior to the start of strength training sessions. The same standardized warm-up was performed before each testing session, which included dynamic stretching and locomotion patterns (i.e., skipping, jogging, and running), and was similar to that of previous literature [17,18,26]. Movement intensities gradually increased over the warm-up duration to prepare subjects for maximal performance during the jump testing. CMJs were performed on the commercially available ForceDecks FD4000 Dual Force Platforms hardware (ForceDecks, London, UK) with a sample rate of 1000 Hz. 

The commercially available ForceDecks software (ForceDecks, London, UK) was used to analyze all the CMJs and generate the CMJ variables using conventional methods [27]. The ForceDecks software uses a 20-N offset from the measured bodyweight, which was quantified before the jump, to define the start of the movement. The end of the eccentric phase and start of the concentric phase was defined as minimum displacement (absolute) which is equal to zero velocity, while take-off was defined as the time point at which the total vertical force fell below the threshold of 20 N below bodyweight. Before calculations were made, the ForceDecks Software combined the data from the two force transducers (sum of the left and right force data). The software uses a 20-N offset from the measured bodyweight obtained prior to the jump to define the start of movement. The end of the eccentric phase and start of the concentric phase was defined as minimum displacement (absolute) which is equal to zero velocity, while take-off was defined as the time point at which the total vertical force fell below the threshold of 20 N below bodyweight. All the asymmetry variables were computed via post hoc analysis following the calculation of traditional CMJ metrics, which was similar to the methods reported in previous literature [21]. 

Descriptions of the CMJ variables are outlined in Table 1. The variables included for analysis were selected because they are part of the ForceDecks Software default asymmetry performance analysis output, and may be of interest to practitioners. A multitude of variables were incorporated in the analysis, because as previous literature has suggested, the most reliable variables may not be the most efficacious in monitoring and detecting neuromuscular fatigue or changes in athlete performance [18].

#### 2.3.2. Countermovement Jump with an Arm Swing (CMJ AS)

Subjects started in the tall standing position, with feet placed hip width to shoulder width apart, but with hands free for movement. Then, the subject was instructed to start with equal weight distribution on both force cells. A visual representation of weight distribution was displayed on a monitor in front of the subject to provide synchronized and integrated feedback, allowing the subject to adjust their positioning for equal quantities of body weight to be distributed on each force cell for the start of the jump. Then, the subject dropped into the countermovement position to a self-selected depth, incorporating an arm swing in their most natural, self-selected manner, followed by a maximal effort vertical jump and landing in an athletic position on the force cells. The subject reset to the starting position after each jump, and the procedure was completed for a total of three jumps. If at any point the subject exhibited excessive knee flexion once airborne, the jump was ruled invalid and repeated. 

#### 2.3.3. Countermovement Jump with No Arm Swing (CMJ NAS)

In the same manner as the CMJ AS, subjects started in the tall standing position, with feet placed hip width to shoulder width apart and hands akimbo. Then, the subject was instructed to start with equal weight distribution on both force cells. A visual representation of weight distribution was displayed on a monitor in front of the subject to provide synchronized and integrated feedback, allowing the subject to adjust their positioning for equal quantities of body weight to be distributed on each force cell for the start of the jump. Then, subjects dropped into the countermovement position to a self-selected depth, followed by a maximal effort vertical jump, and landed in an athletic position on the force cells. The subject would reset to the starting position after each jump, and the procedure was completed for a total of three jumps. If at any point the subject removed their hands from their hips or exhibited excessive knee flexion once airborne, the jump was ruled invalid and repeated.

#### 2.3.4. Inter-Limb Asymmetry Calculation

While several options have been proposed to calculate lower limb asymmetry [28], the purpose of the present analyses was to focus on the variability of the between-limb differences; therefore, the main concern was the absolute difference between the lower limbs for each variable, and was calculated as follows:

Lower-Limb Difference Score = |(Right − Left)|

### 2.4. Statistical Analysis

Data normality was confirmed by the Kolmogorov–Smirnov test, and the results are presented as mean ± SD. The intersession and intrasession mean and reliability were computed for each variable of each limb, as well as for the Lower-Limb Difference Score for each variable of the CMJ when performed with an arm swing, as well as when performed without an arm swing. Relative reliability was assessed using two-way mixed-effects and absolute agreement (3,1) intraclass correlation coefficients (ICC) [29]; furthermore based upon recommendations from prior literature, obtaining an ICC ≥ 0.70 was set as a minimum acceptable reliability [18,19,21,30]. In addition, absolute reliability was assessed using coefficient of variation (CV%) and a typical error of measurement (TE). Acceptable absolute reliability was established at CV ≤ 10% [18,19,21]. Nevertheless, previous literature has suggested that test reliability standards should ultimately be judged by the individual researcher or practitioner based in accordance with their intended use, and that the most reliable variables may not necessarily be the most efficacious in athlete monitoring and performance testing regimens [18]. The typical error of measurement (TE) was calculated by dividing the standard deviation by the square root of two to provide a reflection of the noise within the test caused by biological and technical aspects [31]. In addition, Cronbach’s alpha was computed to assess and provide a measure of internal consistency, allowing the present analysis to be compared with results that only determined Cronbach’s alpha [32]. Lastly, based upon suggestions in previous work, the smallest worthwhile change (SWC) was calculated as 0.2 X between-subject SD, and represented the smallest change that is of benefit for athletic performance [18,19,31]. If the TE ≤ SWC, the metrics were deemed capable of detecting the SWC [18,19,31].

Two-way (Sex [Male versus Female] × Time [Test Session 1 versus Test Session 2]) repeated measures analysis of variance (ANOVA) with Bonferroni post hoc pairwise comparison was used to determine significant sex and time main effects and significant sex by time interactions within the Lower-Limb Difference Score of each variable. Additionally, two-way (Conditions [CMJ NAS versus CMJ AS] × Time [Test Session 1 versus Test Session 2]) repeated measures ANOVA with Bonferroni post hoc pairwise comparison was used to determine significant condition and time main effects and significant condition by time interactions within the Lower-Limb Difference Score of each variable. Effects sizes (Cohen’s *d*) were calculated and interpreted as trivial (0–0.19), small (0.20–0.49), medium (0.50–0.79), and large (0.80 and greater) [33]. SPSS (version 24, Armonk, New York) was used for all the data analysis, with the alpha level set at *p* ≤ 0.05. 

## 3. Results

The results for intersession reliability are outlined in Table 2, while the results of intrasession reliability are in Table 3. During the CMJ AS, 12 of the 16 variables met the acceptable criterion for intersession relative reliability (ICC > 0.70), and six of the 16 variables met the acceptable levels of intersession absolute reliability (CV < 10%). Additionally, 13 of the 16 variables met the acceptable intrasession relative reliability, and 11 of the 16 variables met the acceptable criterion for intrasession absolute reliability, during the CMJ AS. In addition, during the CMJ AS, only Concentric Peak Force, Force at Peak Power, and Take-off Peak Force demonstrated TE ≤ SWC.

During the CMJ NAS, 14 of the 16 variables met the acceptable criterion for intersession relative reliability (ICC > 0.70), and six of the 16 variables met the intersession absolute reliability criterion (CV < 10%). Additionally, 14 of the 16 variables met the acceptable intrasession relative reliability, and 11 of 16 variables met the acceptable criterion for intrasession absolute reliability, during the CMJ NAS. In addition, during the CMJ NAS, only the Concentric Mean Force, Concentric Peak Power, and Force at Peak Power exhibited TE ≤ SWC.

There was a significant Sex X Time Interaction in lower-limb differences for the total net of positive impulse (PosImp) (*p* = 0.005); however, tests of simple effects revealed no significant differences between sex at any time point (*p* > 0.05), which was perhaps due to a small effect or small sample size. There were no other significant Sex X Time Interactions (*p* > 0.05) for any other variable; however, there was a significant Sex main effect for differences in the mean force during the eccentric phase from start of movement to zero velocity (EccMF) between-limb asymmetry (Males = 96.1 ± 11.5 N; Females = 80.9 ± 15.2 N; *p* = 0.003). No significant sex differences in the Lower-Limb Difference Score of any other variable were observed.

There was a significant Condition X Time Interaction [CMJ AS versus CMJ NAS X Testing Session 1 versus Testing Session 2] for the variable of the eccentric rate of force development over first 100-ms epoch of the active braking phase (EccBrakRFD-100). A test of simple effects revealed no differences between conditions during Test Session 1 (CMJ AS = 927.4 ± 134.9 N/s; CMJ NAS = 901.0 ± 141.7 N/s; *p* > 0.05), but a statistically significant difference between condition during Test Session 2 (CMJ AS = 1469.5 ± 274.2 N/s; CMJ NAS = 776.1 ± 158.5 N/s; *p* = 0.003).

As outlined in Table 4, there was a significant condition [CMJ AS versus CMJ NAS] effect, with CMJ AS demonstrating a significant decrease, with a small effect, in lower-limb differences in the total net impulse over first 100-ms epoch of the concentric phase (ConcImp-100) (*p* = 0.048; *d* = 0.40. Additionally, there was a significant decrease, all with small to medium effects, in lower-limb differences during the CMJ NAS in the eccentric rate of force development from minimum force at the start of the active braking phase to zero velocity at the end of the eccentric phase (EccBrakRFD) (*p* = 0.019; *d* = 0.40), EccBrakRFD-100 (*p* = 0.019; *d* = 0.45), eccentric rate of force development from maximum negative velocity to zero velocity at the end of the eccentric phase (EccDecRFD) (*p* = 0.036; *d* = 0.46), and force exerted at peak power (F@PP) (*p* = 0.029; *d* = 0.32). While not statistically significant, there appeared to be a small to medium effect of jumping condition on differences in lower-limb total net impulse over the first 50-ms epoch of the concentric phase (ConcImp-50) (*p* = 0.064; *d* = 0.42), peak force over the concentric phase (ConcPF) (*p* = 0.067; *d* = 0.48), and peak force over the entire take-off phase (Takeoff PF) (*p* = 0.079; *d* = 0.42). In addition, there was no significant time [Test Session 1 versus Test Session 2] effect observed for any variable. 

## 4. Discussion

The purpose of the present study was to establish the intrasession and intersession relative and absolute reliability of variables obtained from a force plate used to quantitate lower extremity inter-limb asymmetry during both the CMJ AS and CMJ NAS. A secondary purpose sought to identify differences in lower extremity inter-limb asymmetries during the CMJ AS compared to the CMJ NAS. The major findings of the present study were (1) the majority of variables met the acceptable criterion for intersession and intrasession relative reliability (ICC > 0.70) during both CMJ protocols; (2) less than half of the variables examined met the acceptable standard for intersession and intrasession absolute reliability (CV < 10%) during both CMJ protocols; (3) only three variables demonstrated the sensitivity to detect the SWC (SWC > TE) during both CMJ protocols; and (4) it appears that the CMJ protocol has an influence on the variability of lower extremity inter-limb asymmetries. 

Previous literature evaluating lower extremity asymmetries have solely focused on outcomes of the concentric phase of the CMJ. In the present study, the concentric force exerted multiplied by time taken (ConcImp), ConImp-50, ConcImp-100, mean force during the concentric phase (ConcMF), ConcPF, and Takeoff PF all demonstrated adequate relative (ICC > 0.800) and absolute reliability (CV < 10%). In parallel to the present study, Menzel et al. [13] tested the reliability of inter-limb asymmetries during the CMJ. While not including an analysis of the eccentric phase, they evaluated symmetries via the lateral symmetry index (LSI), which was calculated as ((value of right limb − value of left limp/greatest value of both limbs) × 100). Their findings included LSI = 5.58%, 23.45%, and 20.66%, for ConcPF, F@PP, and ConcImp, respectively [13]. Additionally, Menzel et al. [13] reported the relative reliability of the CMJ tests during the pilot testing of 16 soccer players (ICC: ConcPF = 0.74, Peak Power = 0.81, and Impulse = 0.71). Using a comparable asymmetries analysis, Benjanvatra et al. [16] identified asymmetry values of ~7–9% for ConcMF, ~8–10% for ConcPF, and ~16–19% for ConcImp, using the CMJ NAS. Meanwhile, Menzel et al. [13] reported similar findings to those of the present study, improvements in both relative and absolute reliability in the present study may relate to our analysis including basketball athletes, where jumping is a large component of their sport, while the aforementioned studies included professional soccer players [13] and recreationally trained subjects [16]. 

Similarly, the present findings support those of Newton et al. [24], which also reported asymmetries of 5.68% in ConcPF and 6.28% in ConcMF, during the CMJ AS. Further corroborating the findings of the present investigation, Impellizzeri et al. [12] examined the reliability of ConcPF during the CMJ NAS, using the unique aforementioned protocol requiring only one force platform. When obtaining an average of three jumps, Impellizzeri et al. [12] reported almost identical findings to that of the present study (TE of 2.8% and an ICC = 0.86). However, the present study adds to the literature by identifying marginal improvements in both the relative and absolute reliability of ConcPF during the CMJ AS when compared to the CMJ NAS, with increases in ICC values, as well as reductions in both CV% and TE. Similarly, Takeoff PF revealed a minor reduction in TE and CV% during the CMJ AS. In contrast, it appears that a marginal improvement in reliability may emerge for ConcImp, ConImp-50, ConcImp-100, and mean force during the concentric phase (ConcMF) during the CMJ NAS. Potential differences in the reliability between the CMJ AS and CMJ NAS may relate to the arm swing more consistently aiding in peak performances captured in an instant or small epoch, such as ConcPF and Takeoff PF, while mild alterations in arm swing synchronization can exacerbate variability among metrics that occur over a longer time frame, such as the total net of positive impulse (PosImp) and ConcMF. In addition, it is important to note that although the present study observed similar inter-limb reliability parameters in regard to ConcMF and ConcPF on average, this does not mean that these metrics can be utilized interchangeably, as previous literature has noted a disagreement in side-to-side differences between the variables [34]. 

EccMF met the accepted reliability criterion during the CMJ NAS, but lacked adequate repeatability during the CMJ AS, while the peak force over the eccentric phase (EccPF) met the relative reliability, but fell just outside the absolute reliability standards established during both CMJ protocols. To our knowledge, this is the first data to identify the reliability of inter-limb EccMF and EccPF during the CMJ, as well as the first to measure the influence of the arm swing. Although variables obtained from the eccentric phase of the CMJ have been implicated as important factors used in injury risk analysis [35,36], the present data would suggest a limitation in using the traditional 10% rule [7,8,9] if EccPF was a variable of interest. Therefore, future literature should build upon the present analysis to evaluate the magnitude of alteration in eccentric components following both acute and chronic fatigue and following injury.

Currently, there is limited evidence assessing the reliability of inter-limb force parameters during the CMJ, with even less specifically identifying the reliability of variables beyond the concentric phase. However, previous literature has examined the reliability of the SLCMJ. Recent work by Bishop et al. [14] examined inter-limb asymmetries in ConcPF and eccentric impulse, as well as concentric impulse during the SLCMJ. Their data illuminate asymmetries ranging from 1.6% to 3.4%, and intra-limb ICC values >0.800 and CV = 3.3–5.8% [14]. These findings corroborate the present data, suggesting that both eccentric and concentric phases yield variables meeting acceptable reliability standards. Similar in the exploration of SLCMJ reliability, data published by Hopper et al. [37] reported a high repeatability (ICC = 0.92) of within-limb flight time during the SLCMJ. More recently, McElveen et al. [38] has also identified a high level of within-limb relative reliability (ICC = 0.850–0.950) of various metrics during the SLCMJ. Similarly, the single leg hop test can be used to assess lower-limb asymmetries. Auggustsson et al. [39] reported values of ICC = 0.98 and CV = 2.5% during a single leg hop protocol comparing the maximal hop length. While these studies examined within-limb reliability, the paralleling inter-limb reliability identified by the present study during the CMJ reinforces previous findings. 

Additionally, in accordance with previous literature examining the traditional combined lower-limb analysis, both F@PP and force at zero velocity—the combined force when velocity is zero/minimum displacement (F@0V) met the acceptable criterion for reliability during both protocols [20,21]. However, PosImp fell just outside the acceptable reliability criterion, and the peak force achieved during the landing phase (Peak Landing Force) lacked reliability during the CMJ AS or the CMJ NAS.

As expected, EccBrakRFD, EccBrakRFD-100, EccDecRFD, and the rate of force development during the landing phase (LandingRFD) variables demonstrated poor relative and absolute reliabilities during both CMJ protocols. These findings are in parallel with previous literature, which has consistently documented “rate” variables as unreliable (CV = 16–50%) [19,21,40,41]. Although the present study used the common recommendation of 1000 Hz, which was thought to be adequate, an increase in the sampling rate may improve the reliability of the RFD indices, while other factors may influence the reliability of the eccentric phase, such as countermovement depth and velocity. Despite the lack of reliability in the present study, RFD parameters have been regarded as vital components in a variety of sports [15,41]. Therefore, future literature may explore increasing the sampling rate in an attempt to improve the reliability of these measures, especially if deemed imperative in the clinical and practical settings. 

During the CMJ AS, only ConcPF, F@PP, and Takeoff PF demonstrated TE ≤ SWC. In contrast, during the CMJ NAS, only ConcMF, ConcPF, and F@PP exhibited TE ≤ SWC. These findings have practical significance, as practitioners should select a CMJ protocol that optimizes the sensitivity of detecting change among the variables they have established as key performance indices for their player assessment and athlete monitoring strategies.

In general, CMJ NAS performance appeared marginally more reliable than the CMJ AS protocol, which may seem surprising, as the CMJ AS is likely more common during sport play, and therefore may be expected to improve familiarity and subsequent repeatability. However, previous literature has observed similar improvements in reliability during CMJ NAS compared to the CMJ AS in other sports requiring high jumping frequency, such as volleyball [42] and basketball [21]. These findings likely indicate that the high degree of sport specificity and frequent repetitions during sport play does not necessarily translate to improvements in reliability over CMJ NAS during testing.

There were no statistically significant differences in the Lower-Limb Difference Scores between sexes, expect for EccMF, which was deemed trivial, as the difference fell at or within the TE (TE: CMJ AS = 26.21 N; CMJ NAS = 21.25 N). Therefore, the sex data was combined to increase the statistical power of the primary analysis directed at examining differences in lower-limb asymmetries between jumping protocols. The present study identified a statistically significant Condition X Time Interaction [CMJ AS versus CMJ NAS X Test Session 1 versus Test Session 2] for the variable of EcceBrakRFD-100. However, this difference is likely not practically significant, and alludes more to the large variability in the metric, as it displayed an intrasession CV = 52% during both CMJ protocols. The present study observed no significant time effect [Test Session 1 versus Test Session 2], which may positively support the reliability of the variables tested in the present study, as they were not statistically different from one testing session to the next. 

A novel finding of the present study was the influence of CMJ protocol on lower-extremity difference scores, as there was a significant condition effect [CMJ AS versus CMJ NAS] with a difference in the lower-extremity difference score between jump protocols for the variables of ConcImp-100, EccBrakRFD, EccDecRFD, and F@0V. While not reaching statistical significance, which was likely due to the small sample size of the present study generating a lack of statistical power, there was also a small to medium effect between jumping conditions for the variables of ConcImp-50, ConcPF, and Takeoff PF. Interestingly, eccentric associated variables appeared to demonstrate a reduction in asymmetry during the CMJ NAS, while concentric variables appeared to demonstrate a reduction in asymmetry during the CMJ AS. These findings suggest that the arm swing influenced CMJ symmetry during the loading phase, but improved asymmetry during propulsion. To the best of our knowledge, there is no previous literature comparing the influence of CMJ protocol on lower-limb asymmetries. Therefore, it is not possible to make a meaningful comparison with previous literature. Regardless, these findings may be relevant and beneficial in guiding coaches and practitioners in selecting a CMJ protocol, once they have established the key performance indices within their athlete monitoring and player assessment strategies. 

The present study lays the foundation for utilizing the CMJ to provide quantitative data to guide return-to-play and return-to-performance protocols following injury. While previous literature has suggested the 10% rule, the present analysis has identified reliable metrics associated with both the concentric and eccentric phases of the CMJ that may prove valuable in enhancing the rehabilitation framework. Interestingly, the authors would like to note that it is plausible that inter-limb variability in asymmetry may be positive, because during injury, it is likely that an athlete would select a movement strategy that would avoid force application to the injured limb, likely manifesting in a consistent asymmetry. Future work should build upon the present findings, characterizing alteration in force outputs during both the concentric and eccentric loading phases of the CMJ following lower-limb injury.

The present study has limitations that warrant discussion. First, although training duration was matched prior to Test Session 1 and Test Session 2, the training load was not monitored with more advanced measures, such as internal or external load-monitoring strategies [17], which may have improved the ability to specifically match basketball-specific training loads prior to testing. Secondly, the present study examined a relatively homogeneous sample of skilled jumpers with limited between-subject variability, which may have contributed to a reduction in relative reliability. Additionally, it should be recognized that the most reliable measures may not be the most efficacious in monitoring changes in fatigue and performance or assessing injury risk parameters. As suggested by previous literature, sport scientists and practitioners ultimately decide upon acceptable reliability criterion and deem a variable that is reliable enough for its intended use [18]. Future research should examine the sensitivity and specificity of the various CMJ asymmetry variables to detect change following fatigue and injury to establish the key performance indices that are useful to clinicians, as well as sports performance practitioners. 

## 5. Conclusions

In conclusion, the findings of the present study offer key practical applications for assessing and monitoring asymmetries in athlete performance. Both the CMJ AS and CMJ NAS provide reliable information with respect to inter-limb asymmetries, suggesting that the CMJ may be a useful tool for performance practitioners and clinicians to effectively monitor the changes associated with performance and injury risk, as well as return-to-play and return-to-performance strategies. Additionally, it appears that the CMJ protocol influences inter-limb asymmetries; therefore, the CMJ testing protocol should be chosen in order to optimize the reliability of the variables of interest to the sport scientist, clinician, or practitioner. 

## Figures and Tables

**Table 1:** CMJ Variable Abbreviations and Definitions.

Variable	Abbreviation	Definition
**Concentric Impulse (Ns)**	ConcImp	Concentric force exerted multiplied by time taken
**Concentric Impulse 50ms (Ns)**	ConcImp-50	Total net impulse over first 50-ms epoch of the concentric phase
**Concentric Impulse 100ms (Ns)**	ConcImp-100	Total net impulse over first 100-ms epoch of the concentric phase
**Concentric Mean Force (N)**	ConcMF	Mean force during the concentric phase
**Concentric Peak Force (N)**	ConcPF	Peak force over the concentric phase
**Eccentric Braking RFD (N/s)**	EccBrakRFD	Eccentric rate of force development from minimum force at the start of the active braking phase to zero velocity at the end of the eccentric phase
**Eccentric Braking RFD-100ms (N/s)**	EccBrakRFD-100	Eccentric rate of force development over first 100-ms epoch of the active braking phase
**Eccentric Deceleration RFD (N/s)**	EccDecRFD	Eccentric rate of force development from maximum negative velocity to zero velocity at the end of the eccentric phase
**Eccentric Mean Force (N)**	EccMF	Mean force during the eccentric phase from start of movement to zero velocity
**Eccentric Peak Force (N)**	EccPF	Peak force over the eccentric phase
**Force at Peak Power (N)**	F@PP	Force exerted at peak power
**Force at Zero Velocity (N)**	F@0V	Combined force when velocity is zero/minimum displacement
**Landing RFD (N/s)**	LandingRFD	Rate of force development during the landing phase
**Peak Landing Force (N)**	Peak Landing Force	Peak force achieved during the landing phase
**Positive Imp (Ns)**	PosImp	Total net of positive impulse
**Take-Off Peak Force (N)**	Takeoff PF	Peak force over the entire take-off phase

**Table 2:** CMJ Asymmetry Intersession Reliability During the CMJ AS and NAS.

	Intersession Reliability											
	Arm Swing (CMJ AS)						No Arm Swing (CMJ NAS)					
CMJ Variable	Mean ± SD	Cron	ICC	SWC	TE	CV%	Mean ± SD	Cron	ICC	SWC	TE	CV%
**Conc Imp (Ns)**	263.0 ± 25.5	0.973	0.747	12.89	18.03	9.1	242.2 ± 14.5	0.987	0.868	8.44	10.22	6.1
**Conc Imp-50ms (Ns)**	44.0 ± 4.7	0.980	0.800	2.28	3.32	11.2	48.3 ± 4.6	0.984	0.839	2.32	3.24	9.9
**Conc Imp-100ms (Ns)**	91.0 ± 9.7	0.978	0.786	4.74	6.87	11.3	97.0 ± 8.0	0.986	0.854	4.34	5.64	8.7
**Conc Mean Force (N)**	916.6 ± 52.6	0.989	0.879	32.76	37.23	5.8	878.4 ± 45.2	0.992	0.909	31.11	31.99	5.3
**Conc Peak Force (N)**	1201.3 ± 52.6	0.995	0.942	45.00	37.17	4.5	1084.8 ± 58.3	0.992	0.911	41.24	41.21	5.4
**Ecc Brak RFD (N/s)**	2609.5 ± 659.4	0.976	0.772	293.98	466.29	27.9	2578.4 ± 618	0.982	0.823	293.17	436.98	26.7
**Ecc Brak RFD-100ms (N/s)**	2137.1 ± 1238.5	0.834	0.296	338.03	875.75	58.1	1548.6 ± 815.9	0.918	0.482	266.32	576.93	54.5
**Ecc Dec RFD (N/s)**	2945.3 ± 802.1	0.981	0.851	395.93	567.15	32.3	3240.3 ± 812.8	0.985	0.844	425.77	574.73	28.3
**Ecc Mean Force (N)**	442.3 ± 37.1	0.956	0.644	13.43	26.21	8.4	441.4 ± 30.1	0.972	0.744	12.97	21.25	6.8
**Ecc Peak Force (N)**	918.9 ± 97.8	0.98	0.803	47.06	69.13	11.1	978.0 ± 105.6	0.981	0.815	49.38	74.64	11.1
**Force at Peak Power (N)**	1096.2 ± 47.2	0.995	0.940	39.09	33.38	4.4	958.8 ± 47.5	0.991	0.901	30.51	33.58	5.0
**Force at Zero Vel (N)**	893.6 ± 93.6	0.980	0.805	46.4	66.22	10.9	974.4 ± 105.7	0.981	0.812	49.11	74.72	11.1
**Landing RFD (N/s)**	4,3754.7 ± 1,4970.4	0.956	0.647	6350.06	10585.67	34	4,2511.3 ± 1,3481.2	0.97	0.729	5895.85	9532.61	33.8
**Peak Landing Force (N)**	2524.4 ± 514.3	0.970	0.730	218.77	363.64	20.7	2480.7 ± 499.5	0.97	0.733	201.28	353.23	21.5
**Positive Imp (Ns)**	735.2 ± 82	0.933	0.538	26.01	57.96	11.0	706.4 ± 74.9	0.922	0.498	22.13	52.96	10.7
**Take-Off Peak Force (N)**	1204.3 ± 53.9	0.994	0.937	44.52	38.14	4.6	1086.9 ± 58.4	0.992	0.911	41.36	41.3	5.4

*CMJ represents countermovement jump; AS represents arm swing; NAS represents no arm swing; SD represents standard deviation; Cron represents Cronbach’s alpha; ICC represents intraclass correlation coefficient; TE represents typical error; SWC represents smallest worthwhile change; CV% represents coefficient of variation.

**Table 3:** CMJ Asymmetry Intrasession Reliability During the CMJ AS and CMJ NAS.

	Intrasession Reliability											
	Arm Swing (CMJ AS)						No Arm Swing (CMJ NAS)					
CMJ Variable	Mean ± SD	Cron	ICC	SWC	TE	CV%	Mean ± SD	Cron	ICC	SWC	TE	CV%
**Conc Imp (Ns)**	263.0 ± 19.7	0.964	0.817	12.76	13.94	7.1	242.1 ± 13.1	0.979	0.886	8.45	9.25	5.5
**Conc Imp-50ms (Ns)**	44.0 ± 4	0.967	0.832	2.28	2.84	9.6	48.3 ± 4.1	0.974	0.863	2.32	2.91	8.9
**Conc Imp-100ms (Ns)**	91.0 ± 8.4	0.962	0.809	4.74	5.92	9.7	97.0 ± 7.1	0.978	0.932	4.34	5.00	7.7
**Conc Mean Force (N)**	916.6 ± 47.9	0.979	0.884	32.81	33.84	5.3	878.4 ± 41	0.986	0.923	31.14	29.01	4.8
**Conc Peak Force (N)**	1201.3 ± 46.3	0.992	0.95	45.08	32.76	4.0	1084.8 ± 54.3	0.985	0.918	41.28	38.39	5.1
**Ecc Brak RFD (N/s)**	2609.5 ± 581.5	0.961	0.805	294.41	411.18	25.1	2578.4 ± 548.5	0.971	0.849	293.65	387.86	23.9
**Ecc Brak RFD-100ms (N/s)**	2137.1 ± 1112	0.771	0.363	333.66	786.3	52.0	1548.6 ± 779.7	0.842	0.472	263.48	551.36	51.5
**Ecc Dec RFD (N/s)**	2945.3 ± 684.6	0.971	0.851	396.59	484.07	27.3	3240.3 ± 721.9	0.976	0.872	426.49	510.44	25.4
**Ecc Mean Force (N)**	442.3 ± 36.7	0.906	0.615	13.45	25.95	8.3	441.4 ± 29.8	0.940	0.726	12.99	21.09	6.7
**Ecc Peak Force (N)**	918.9 ± 86	0.969	0.838	47.07	60.82	9.8	978.0 ± 94.6	0.970	0.842	49.45	66.92	10.0
**Force at Peak Power (N)**	1096.2 ± 43.6	0.991	0.948	39.16	30.84	4.1	958.8 ± 40.8	0.987	0.925	30.56	28.87	4.3
**Force at Zero Vel (N)**	893.6 ± 81.6	0.969	0.839	46.41	57.73	9.6	974.4 ± 95	0.969	0.840	49.19	67.20	10.1
**Landing RFD (N/s)**	43754.7 ± 1,2295.8	0.947	0.751	6347.75	8694.47	28.3	42511.3 ± 1,1777.2	0.953	0.774	5899.38	8327.73	29.9
**Peak Landing Force (N)**	2524.4 ± 465.5	0.953	0.772	218.97	329.16	19.2	2480.7 ± 459.6	0.952	0.768	201.66	325.02	19.9
**Positive Imp (Ns)**	735.2 ± 77.6	0.874	0.538	26.04	54.89	10.3	706.4 ± 71.3	0.868	0.523	22.14	50.39	10.2
**Take-Off Peak Force (N)**	1204.3 ± 47.5	0.991	0.947	44.59	33.58	4.1	1086.9 ± 54.6	0.986	0.918	41.40	38.62	5.1

*CMJ represents countermovement jump; AS represents arm swing; NAS represents no arm swing; SD represents standard deviation; Cron represents Cronbach’s alpha; ICC represents intraclass correlation coefficient; TE represents typical error; SWC represents smallest worthwhile change; CV% represents coefficient of variation.

**Table 4:** Differences in Inter-Limb Asymmetries During the CMJ AS compared to the CMJ NAS.

Variable	CMJ AS	CMJ NAS
**Conc Imp (Ns)**	14.5 ± 9.0	15.2 ± 6.4
**Conc Imp-50ms (Ns)**	3.7 ± 1.7	4.4 ± 2.1
**Conc Imp-100ms (Ns)**	6.5 ± 3.2	7.8 ± 3.2 *
**Conc Mean Force (N)**	50.6 ± 28.2	54.8 ± 22.3
**Conc Peak Force (N)**	47.0 ± 27.5	60.1 ± 32.4
**Ecc Brak RFD (N/s)**	507.1 ± 289.2	393.4 ± 199.0 *
**Ecc Brak RFD-100ms (N/s)**	1198.4 ± 803.2	838.5 ± 639.6
**Ecc Dec RFD (N/s)**	632.3 ± 280.4	502.1 ± 252.6 *
**Ecc Mean Force (N)**	57.3 ± 30.0	48.5 ± 19.8
**Ecc Peak Force (N)**	85.3 ± 39.5	95.9 ± 52.0
**Force at Peak Power (N)**	84.0 ± 37.7	96.5 ± 52.3
**Force at Zero Vel (N)**	46.7 ± 26.3	38.4 ± 25.2 *
**Landing RFD (N/s)**	13133.5 ± 9472.6	11727.2 ± 7779.9
**Peak Landing Force (N)**	547.3 ± 277.8	490.3 ± 196.5
**Positive Imp (Ns)**	100.4 ± 52.5	94.1 ± 37.3
**Take-Off Peak Force (N)**	48.5 ± 29.3	60.7 ± 32.0

CMJ represents countermovement jump; AS represents arm swing; NAS represents no arm swing; Data presented as mean ± standard deviation; * represents *p* < 0.05.
